# Patent Medicine

**Published:** 1913-09

**Authors:** E. H. Golar

**Affiliations:** Chemist Food and Drug Department, State of Texas


### Patent Medicine

BY E. H. GOLAR, CHEMIST FOOD AND DRUG DEPARTMENT, STATE OF TEXAS

Some day the history of what is known as Patent Medicine will be written—the history of a new industry, born in the years of 1880, dying, but dying hard in the years of 1920. It will be described by disinterested writers as the one of the parasites of modern science thriving upon our new method of communication, upon rapid and cheap traveling, upon the wonderful growth of newspapers, periodicals, and magazines.

Every civilization, every age, every century gives us the history of quackery, preying upon the ignorance, the fears, the desires of man. Religion and medicine are the richest field exploited for the purpose of obtaining tangible gifts, goods, or money in exchange for intangible services during or after life.

Before the advent of railroads, newspapers, telegraph, telephone, the quack was forcibly a local artist. His fame and wares could not extend over very wide boundaries. When these boundaries fell before the discoveries and endeavor of science, the local quack of ability had now the world thrown open for his activities. The small, petty and local frauds perpetrating upon an ignorant and credible public became monumental impositions compared to which the ingenuity of a "Get-Rich-Wallingford" is child play; gold brick schemes, hoenst; Mexican mining stock, secure; and lots in Heaven, truthful.

Ignorance, cupidity can be preyed upon without exciting much pity, but it is time to open our eyes on a business that capitalize the tears of a mother, or the sufferings of a fellowman. Space does not allow me to review the history of the fight of the National Food and Drugs Act with some of our best known patent medicine frauds from Antikamnia, Peruna, Piso's Cure, Bromidia, Bromo-Seltzer, Capudine, Manola, Buffalo Lithia Water, to Madam Yale's Excelsior Complexion Bleach.

The pure food laws have done wonders in forcing most of the preparations now on the market to bear a label conforming to the truth. But if the label is correct, the Food and Drug Department, however, is unable to regulate the absurd advertisement that is attached to it. The facilities for advertisements of this kind, offered by a venal press on a vaster scale that has ever been known before help to keep quackery alive and to delude incalcuble numbers of victims whose faith to the promise of cure so impudently put forward leads to the loss, not only of money but of health. We believe that if newspapers, says the *British Medical Journal*, refuse to publish such advertisements quackery would receive its death blow. Americans hope to be made sober by acts of legislature, but they would resent very keenly a law preventing them from taking any pills they find good, or rubbing themselves with some nondescript, "Mother's Friend". But the law should see to it that the people are not misled by fraudulent statements.. If such statements are punishable when made in the prospectus of a company, it is surely not less important that they should be penalized when made in advertisements which promise relief to hopeless suffering. It is the quack advertisement with its suggestion of ill-health, reinforced by the enumeration of symptoms which, even when they exist, have not necessarily any sinister significance, that prepares the mind of the reader for the reception of the insinuated falsehood. It is a danger even to the healthy, for it tends to engender self-consciousness, which is the root of innumerable evils. But to sufferers from diseases for which medicine at present can only offer doubtful remedies, it is subtle poison.

It would require a volume to take one by one the different patent medicine frauds now imposing themselves upon the public, and possibly I could best quote here the words of Samuel Hopkins Adams, when in his book, "The Great American Fraud," published under the auspices of the American Medical Association, he says:

"It is impossible, even in a series of articles to attempt more than an exemplary treatment of the patent-medicine frauds. The most degraded and degrading, the 'lost vitality' and 'blood disease' cures, reeking of terrorization and blackmail,, can not from their very nature be treated of in a lay journal. Many dangerous and health-destroying compounds will escape through sheer inconspicuousness. I can touch on only a few of those which may be regarded as typical: the alcoholic stimulators, as represented by Peruna, Paine's Celery Compound and Duffy's Pure Malt Whiskey (advertised as an exclusively medical preparation) ; the catarrh powders, which breed cocaine slaves, and the opium-containing soothing syrups which stunt or kill helpless infants; the consumption cures, perhaps the most devilish of all, in that they destroy hope where hope is struggling against bitter odds for existence; the headache powders, which enslave so insidiously that the victim is ignorant of his own fate; the comparatively harmless fake as typified by that marvelous product of advertising effrontery, Liquizone; and, finally, the system of exploitation and testimonials on which the whole vast system of bunco rests, as on a flimsy but cunningly constructed foundation."

